# Significant Modulation of Vortex Resonance Spectra in a Square-Shape Ferromagnetic Dot

**DOI:** 10.3390/nano12132295

**Published:** 2022-07-04

**Authors:** Shaojie Hu, Xiaomin Cui, Kang Wang, Satoshi Yakata, Takashi Kimura

**Affiliations:** 1 Center for Spintronics and Quantum Systems, State Key Laboratory for Mechanical Behavior of Materials, School of Materials Science and Engineering, Xi’an Jiaotong University, Xi’an 710049, China; shaojiehu@mail.xjtu.edu.cn (S.H.); 3120102052@stu.xjtu.edu.cn (K.W.); 2 Department of Physics, Kyushu University, 744 Motooka, Fukuoka 819-0395, Japan; 3 School of Physical Science and Technology, Northwestern Polytechnical University, Xi’an 710129, China; 4 Department of Information Electronics, Fukuoka Institute of Technology, 3-30-1 Wajiro-higashi, Higashi-ku, Fukuoka 811-0295, Japan; yakata@fit.ac.jp

**Keywords:** magnetic vortex, vortex core resonance, spin dynamics, patterned ferromagnetic structure

## Abstract

The resonance property of a magnetic vortex contained within a micron-sized square Py dot was detected using an amplitude-modulated magnetic field excitation technique. We found a significant modulation of the resonant spectra as the external magnetic field changes. The Lorentzian-like spectrum changes from a peak to a dip via a transition of anti-Lorentzian-like spectra. By conducting the micromagnetic simulations, we confirmed that the transition behavior results from the unusual resistance change depending on the vortex core center position. Additionally, the power dependence of the anti-Lorentzian-like spectra revealed a fairly persistent coexistence of peak and dip. Thus, the tunable spectra suggest one way to develop an integratable radiofrequency microcircuits.

## 1. Introduction

The dynamic properties of magnetic materials or devices are interesting research areas because of their vast frequency tuning range from several megahertz to gigahertz and even terahertz [1,2,3,4]. The total energy of a ferromagnetic unit is determined by the competition among the exchange field, demagnetization field, external field and anisotropic field etc. [5]. Studies of ferromagnetic resonance or spin waves often focus on ferromagnetic materials with an external field that exceeds the anisotropic field and demagnetization field [2]. However, at low magnetic fields, the magnetic momentum distribution becomes nonuniform due to the competition between the anisotropic field and demagnetization field, which depend on the symmetry of the crystal structure and geometry, respectively. The complex magnetization distribution complicates magnetic dynamics analysis. The magnetic vortex, a topological structure defined by in-plane curling magnetization and out of core magnetization, has piqued the interest of researchers owing to its exceptional thermal stability [6,7,8]. The vortex core could be triggered to gyroscopic motion around its equilibrium point with a sub-gigahertz resonant frequency [9,10,11,12]. The study of vortex core dynamics would contribute to a better understanding of vortex resonant modes and the underlying physics, hence enabling the creation of vortex-based oscillators or filters. Much research has been conducted to investigate magnetic vortex dynamics in circular ferromagnetic disks [1,13,14,15,16,17,18,19,20,21,22,23], thanks to the simplified analysis in their perfect symmetric structures.

As is well-known, one effective way to manipulate the magnetic vortex property is to alter the geometry of the confined structure. A prior study demonstrated that asymmetric nucleation energy can be used to alter the chirality of an odd-sided polygon [24]. Additionally, the triangular dot with a magnetic vortex has been confirmed to have wide tunability with the external field [25,26]. Moreover, well-defined magnetic vortex excitation in square elements has been obtained by measuring the induction voltage while applying an external field along the edge [11,27]. To broaden our understanding, we applied a sensitive electrical measurement approach with the separation between the excitation and detection circuits that enables the neglect of complex analysis and the access to dynamics under high power excitation [28]. Therefore, in this study, we investigated the dynamics of a magnetic vortex confined in a chain of square ferromagnetic dots using an amplitude-modulated magnetic field excitation technique under a variety of external magnetic fields. The dynamic response spectrum transitioned from a Lorentzian-like shape to an anti-Lorentzian-like shape as the external field was changed. The transition process was thoroughly investigated and verified by conducting a micromagnetic simulation. Moreover, the power dependence of the anti-Lorentziant-like spectra has also been investigated.

## 2. Result and Discussion

**Figure 1:**
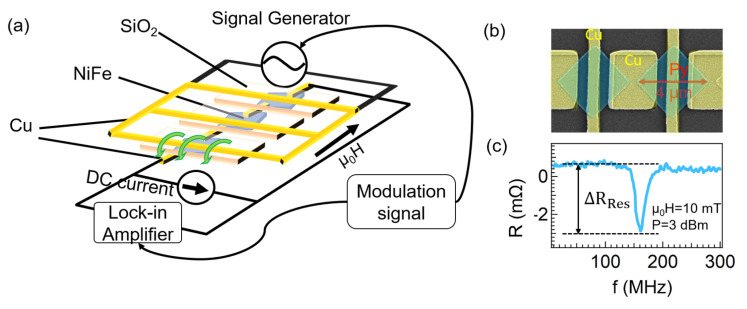
(**a**) Schematic measurement setup for the detection of magnetic vortex dynamics. An in-plane static magnetic field ($\mu_{0}H$) is applied parallel with the chain of the Py dots. The amplitude-modulated RF field is injected from the periodically patterned Cu electrodes into the Py dots. The voltage is detected by flowing DC current in another separated circuit using a lock-in measurement system. (**b**) SEM image for the device with the diagonal distance of 4 μm and center-center distance of 4.4 μm of the square disk. (**c**) A representative spectrum as a function of the input RF frequency under a magnetic field of 10.0 mT. $\Delta R_{\mathrm{Res}}$ is the resistance change between the oscillation and non-oscillation states.

A chain of square-shaped ferromagnetic dots was fabricated on Si substrate using conventional lift-off and electron beam lithography techniques. Here, a 40-nm-thick permalloy (Py) film was evaporated by electron-beam evaporation under the pressure of $2\times 10^{-7}$ Pa. The square device with a diagonal distance of 4 μm was fabricated. The diagonal distance corresponds to the diameter of the circumscribed circle of the square dot. Besides, the center-center distance was designed as 1.1 times of the diagonal distance. The Py dots were connected by Cu pads, and the microwave signal was injected into the Py dots by periodical Cu electrodes on top of the Py dots. Cu pads and electrodes with a thickness of 200 nm were deposited by a Joule heat evaporator after surface cleaning of the Py dots under low-energy Ar ion milling. In addition, ${\mathrm{SiO}}_{2}$ was prepared to cut the connection between Py dots and Cu electrodes. As shown in the SEM image of part of the device in Figure 1b, one of the diagonal lines of the square is parallel to the chain of the Py dots.

To explore the dynamic properties of the magnetic vortices confined in square Py dots, a sensitive detection technique was employed and the schematic circuit is shown in Figure 1a. In the measurement, an amplitude-modulated RF signal was applied to the Cu electrode to oscillate the vortex core confined in the square dot. Simultaneously, the voltage response of the magnetic vortices was detected by another separated circuit combining the lock-in measurement system with a DC current flowing in the chain of Py dots. This separation between the excitation and detection line enables the simplification of the complex analysis. The dynamical properties of the magnetic vortices were observed by sweeping the RF frequency, while a static magnetic field was applied along the chain. As seen in Figure 1c, a typical spectrum with a resonant dip at 161.0 MHz was obtained at the static field of 10.0 mT. $\Delta R_{\mathrm{Res}}$, the difference between the baseline and the resonant dip, represents the magnetoresistance change between the oscillation and non-oscillation states of the magnetic vortices.

**Figure 2:**
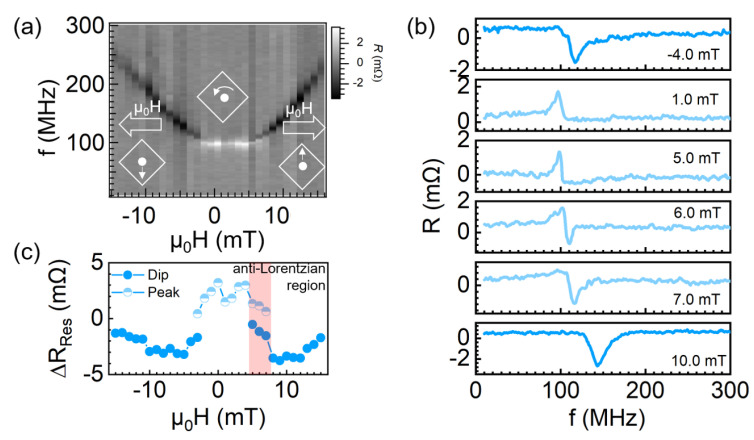
(**a**) The image plot of frequency dependent spectra with sweeping the external magnetic field. The vortex core displacement is also shown with counterclockwise chirality. (**b**) Magnetoresistance spectra with specific external magnetic fields. (**c**) $\Delta R_{\mathrm{Res}}$ as a function of the magnetic fields. The blue and light blue dots stand for the resonant dip and peak, respectively.

The resonant behavior was carefully investigated by changing the magnetic field. Figure 2a shows the image plot of frequency dependent spectra with sweeping the external magnetic field at RF power 3 dBm. First, we could see the resonant frequency varies from 98.0 MHz to 238.0 MHz, which indicates the achievement of a large modulation of the resonant frequency. At the low field region, the almost level white line indicates spectra have resonant peaks. However, the monotonically increased black line indicates the spectra have resonant dips in such field region. To clarity how does the transition of spectra, we also plot some spectra at specific fields in Figure 2b. Interestingly, we observed an significant anti-Lorentzian-like spectrum at 6.0 mT, where a resonant dip appears at 118.0 MHz besides the resonant peak of 98.0 MHz. The amplitude of the resonant peak is almost same as the resonant dip in this field. The resonant dip became stronger and dominant with the increase of field. Finally, it becomes a single resonant dip again when the magnetic field is larger than 7.0 mT. The experimental observation of the coexistence of both the oscillation peak and dip is unique. Usually, two oscillation responses (peak or dip) correspond to two oscillation states according to a previous study [29]. To clarify, we conducted a micromagnetic simulation to check whether these two states represent two oscillation modes or not.

**Figure 3:**
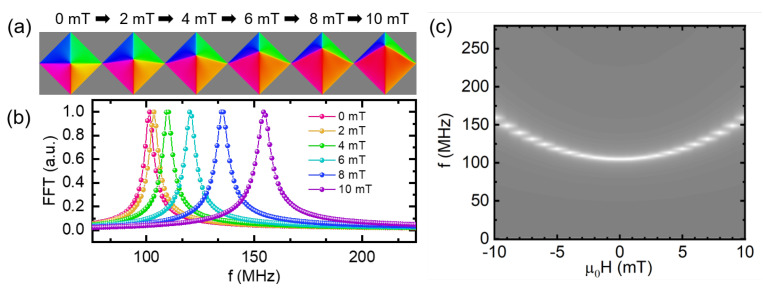
(**a**) Simulated domain structures of the magnetic vortex under various external magnetic fields. (**b**) Field dependence of the FFT spectra under the partial positive magnetic fields. (**c**) Image plot of the FFT spectra under the external magnetic from −10.0 mT to 10.0 mT.

Here, we performed the micromagnetic simulation using MuMax${}^{3}$ [30]. The computational structure was designed with the same size and thickness as the fabricated Py dot. The domain was discretized into mesh sizes of 4 nm × 4 nm × 40 nm. We used the typical microstructural parameters of Permalloy, with an exchange stiffness constant $A_{\mathrm{ex}}=1.3\times 10^{-11}$J/m, damping parameter α=0.006, saturation magnetization $M_{s}=8\times 10^{5}$A/m, and zero magnetocristalline anisotropy constant. During the simulation, we assumed that the chirality is counterclockwise and the polarity points vertically up of the plane. First, we obtained the in-plane components of the magnetization distribution M confined in the square dot under various external fields parallel to the diagonal direction, as shown in Figure 3a. It is natural to see the vortex core shifts up under a positive field in the x-direction.

To obtain the resonant spectra, a sinc based exciting field, $h\left(t\right)=h_{0}\mathrm{sinc}\left(2\pi f_{c}\left(t-t_{0}\right)\right){\hat{e}}_{z}$, with $\mu_{0}h_{0}$ = 10 mT, $f_{c}$ = 50 GHz, $t_{0}$ = 5 ns, is applied locally in the wave guide. Based on the present magnetization profile, we calculated the field dependence of the FFT spectra of magnetic momentum for vortex-core gyrations as shown in Figure 3b. The resonant frequencies are within the same range as the experimental results. For further comparison, we also give the image plot of the frequency spectra for the external field in Figure 3c. The continued white line indicates that each spectrum only has one resonant peak in all field regions. This implies that there should be just one oscillation mode, which would not explain the experimental spectrum transition.

To gain insight into the observed unique spectra, the dynamic processes were further studied by calculating the corresponding magnetoresistance in the Py dot. With assuming the current along the x direction, we can use the magnetization direction of each unit cell to calculate its resistivity using the anisotropic magnetoresistance (AMR) effect. The AMR of Py is given by the following equation:

(1) $$\rho =\rho_{0}\left(1+\chi cos^{2}\theta \right)$$

where, $\rho_{0}$, the resistivity of Py, is approximately 3.6$\times 10^{8}\;\Omega \cdot m$. Based on our experimental results, the AMR ratio χ is about 1.4%. θ is the angle between the magnetization and the current direction. Finally, the total resistance was calculated by connecting all cell resistance in parallel and series for each magnetic states.

**Figure 4:**
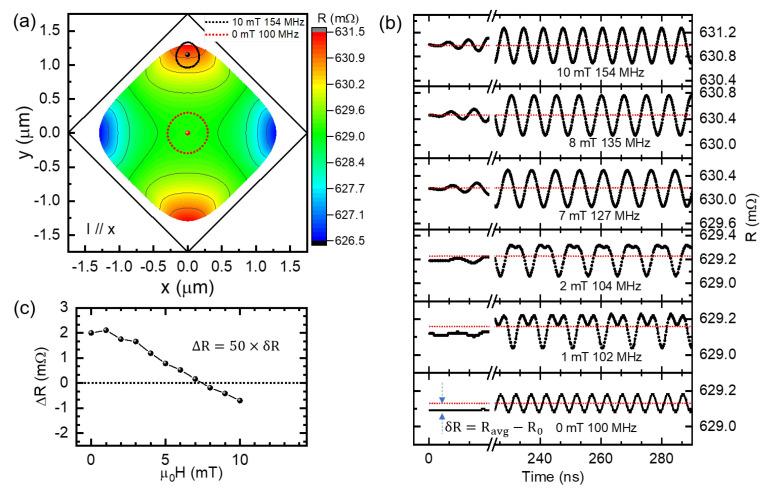
(**a**) Contour map of magnetoresistance in the square Py dot as a function of the vortex core center position. The magnetoresistance was calculated for current parallel to x direction. Besides, the core trajectories of resonant state are plotted as the red dotted line and black dotted line under 0 mT and 10.0 mT, respectively. (**b**) The numerically calculated time dependence of anisotropic magnetoresistance under the resonant state with the various external fields. (**c**) The calculated magnetoresistance change ΔR for 50 square dots with respect to the magnetic field.

Figure 4a provides a contour plot of the magnetoresistance as a function of the vortex core position in the square dot. It’s clearly to see the homogeneously distributed magnetoresistance with two-fold symmetry. The magnetoresistance is largest at the maximum y value, whereas it is smallest at the maximum x value. Besides, the vortex core trajectory is circular shape at zero field during oscillation state. However, the core trajectory deviates from circular and the radius of the core motion decreases under 10.0 mT. It appears that the shape distorts significantly when the vortex core moves to the top. This should result from a larger restriction of the potential when the vortex core approaches the upper vertex. Correspondingly, the resonant frequency increases from zero field to 10.0 mT.

In addition, we plotted the time dependent resistance at resonant frequency under some of the magnetic fields in Figure 4b. We also calculated the average resistance after the stable oscillation in ten periods and marked it as red dashed lines. By calculating the difference between the resistance in the non-oscillation and oscillation states, we obtained δR which can response the oscillation states. For comparing with the experimental results, we also plotted ΔR of 50 square dots as a function of the external field as shown in Figure 4c. The calculated value agrees with the experimental result within the same order of magnitude. Moreover, ΔR first increases with the external field and then decreases to the negative value. This tendency is also in good agreement with the experimental results shown in Figure 2c. To understand this behavior, we checked the time dependence of the resistance curve in Figure 4b. We can notice that the two-fold symmetry resistance response is broken when the field is away zero. Finally, only one-fold symmetry resistance response appears. During the transition, the significant deviation from the sine function of the curve indicates an unusual magnetoresistance change under this field during oscillation. So, the highest δR value was observed around 1.0 mT based on the numerical results in Figure 4c. This feature was also clearly observed in experimental results shown in Figure 2c.

**Figure 5:**
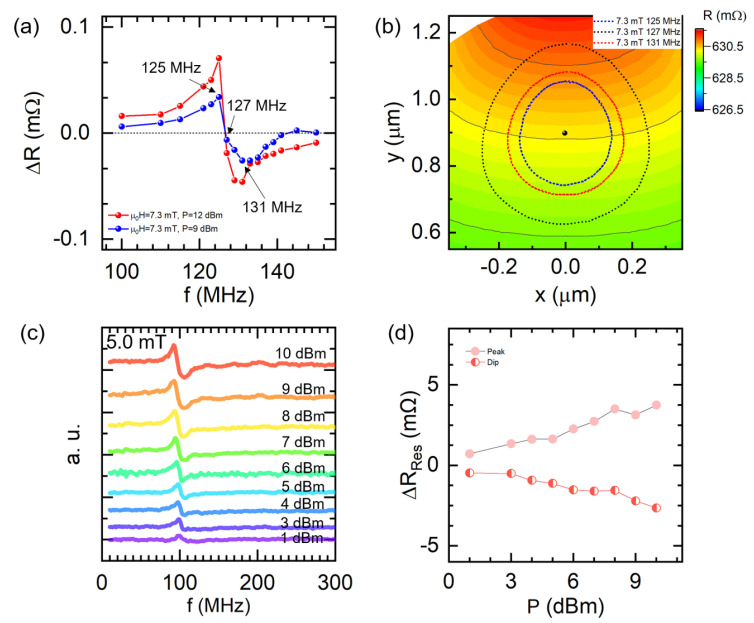
(**a**) The numerical resistance change as a function of the excitation frequency with the magnetic field of 7.3 mT at 9 and 12 dBm, respectively. (**b**) Simulated core trajectories at excitation frequency of 125 MHz, 127 MHz, and 131 MHz with the same magnetic field of 7.3 mT and RF power of 9 dBm. (**c**) The experimental spectra obtained with the magnetic field of 5.0 mT at various RF power. (**d**) $\Delta R_{\mathrm{Res}}$ as a function of the RF power; The red dot and red half-filled dot represent the value of the resonant peak and resonant dip, respectively.

As for the experimentally observed anti-Lorentzian-like spectra, we think that it should match a special magnetic field with both δR>0 and δR<0. According to Figure 4c, the ΔR value for 7.0 mT is positive, whereas that for 8.0 mT is negative. Correspondingly, we predicted this special situation located in the field range between 7.0 mT and 8.0 mT. Therefore, we further studied the time-dependent resistance for the field between 7.0 mT and 8.0 mT and calculated the δR with respect to the RF frequency. As shown in Figure 5a, we successfully obtained the special spectra with both the peak and dip at the magnetic field of 7.3 mT. Besides, we plotted the core trajectories of different frequencies under 7.3 mT. As seen in Figure 5b, the trajectory radius of 127.0 MHz is the largest compared with that of 125.0 MHz and 131.0 MHz. Those results indicate that the resonant frequency should be 127.0 MHz at 7.3 mT, even the corresponding ΔR is almost zero. Therefore, the experimentally obtained resistance response of the peak and dip in the spectrum should be attributed to the unusual magnetoresistance change of the core position during oscillation, rather than the two oscillation modes. Namely, the anti-Lorentzian resistance curve with the peak and dip is an extrinsic behavior of one vortex oscillation mode. By examining the simulated spectra obtained under 9 dBm and 12 dBm, we find the peak and dip are still very stable. In addition, ΔR increases with an increase in RF power.

To validate this simulation result, we evaluated the dynamical behavior with a magnetic field of 5.0 mT under different RF power as shown in Figure 5c. Clearly, the double resonance responses of the device exist consistently from 1 dBm to 10 dBm, demonstrating that the anti-Lorentz-like spectra are very stable with increasing RF power. We also summarized the $\Delta R_{\mathrm{Res}}$ for both the resonant peak and dip with respect to the RF power, as shown in Figure 5d. Both $\Delta R_{\mathrm{Res}}$ monotonically increase, which is in good agreement with the simulation. Moreover, it seems that the upside resonant peak becomes weaker while the resonant dip becomes stronger and dominant with the power increase at 5.0 mT. It may be related to the slight resonant frequency modulation under higher excitation power.

## 3. Conclusions

We experimentally explored the resonant properties of a chain of square-shaped Py dots. A large modulation has been achieved not only for the resonant frequency but also for the shape of the resonance spectra. The spectra changes from a peak to a dip via an anti-Lorentzian transition with the external magnetic field. We confirmed that the oscillation peak and dip should be attributed to the unusual resistance change depending on the core-center position rather than two oscillation modes using the micromagnetic simulation. They may find an use in signal processing based on microwave logic circuits.

## Data Availability

The data supporting the findings of this study are available from the corresponding author upon reasonable request.
